# HexSDF Is Required for Synthesis of a Novel Glycolipid That Mediates Daptomycin and Bacitracin Resistance in C. difficile

**DOI:** 10.1128/mbio.03397-22

**Published:** 2023-02-14

**Authors:** Anthony G. Pannullo, Ziqiang Guan, Howard Goldfine, Craig D. Ellermeier

**Affiliations:** a Department of Microbiology and Immunology, Carver College of Medicine, University of Iowa, Iowa City, Iowa, USA; b Department of Biochemistry, Duke University Medical Center, Durham, North Carolina, USA; c Department of Microbiology, Perelman School of Medicine, University of Pennsylvania, Philadelphia, Pennsylvania, USA; d Graduate Program in Genetics, University of Iowa, Iowa City, Iowa, USA; Texas A&M University; University of Oklahoma Health Sciences Center

**Keywords:** two-component regulatory system, cell envelope, signal transduction, gene expression, glycolipid synthesis, membrane biogenesis, glycolipids

## Abstract

Clostridioides difficile is a Gram-positive opportunistic pathogen responsible for 250,000 hospital-associated infections, 12,000 hospital-associated deaths, and $1 billion in medical costs in the United States each year. There has been recent interest in using a daptomycin analog, surotomycin, to treat C. difficile infections. Daptomycin interacts with phosphatidylglycerol and lipid II to disrupt the membrane and halt peptidoglycan synthesis. C. difficile has an unusual lipid membrane composition, as it has no phosphatidylserine or phosphatidylethanolamine, and ~50% of its membrane is composed of glycolipids, including the unique C. difficile lipid aminohexosyl-hexosyldiradylglycerol (HNHDRG). We identified a two-component system (TCS), HexRK, that is required for C. difficile resistance to daptomycin. Using transcriptome sequencing (RNA-seq), we found that HexRK regulates expression of *hexSDF*, a three-gene operon of unknown function. Based on bioinformatic predictions, *hexS* encodes a monogalactosyldiacylglycerol synthase, *hexD* encodes a polysaccharide deacetylase, and *hexF* encodes an MprF-like flippase. Deletion of *hexRK* leads to a 4-fold decrease in daptomycin MIC, and that deletion of *hexSDF* leads to an 8- to 16-fold decrease in daptomycin MIC. The Δ*hexSDF* mutant is also 4-fold less resistant to bacitracin but no other cell wall-active antibiotics. Our data indicate that in the absence of HexSDF, the phospholipid membrane composition is altered. In wild-type (WT) C. difficile, the unique glycolipid HNHDRG makes up ~17% of the lipids in the membrane. However, in a Δ*hexSDF* mutant, HNHDRG is completely absent. While it is unclear how HNHDRG contributes to daptomycin resistance, the requirement for bacitracin resistance suggests it has a general role in cell membrane biogenesis.

## INTRODUCTION

Clostridioides difficile is a Gram-positive obligate anaerobe and opportunistic pathogen. C. difficile causes approximately 220,000 hospital-associated infections, leading to about 12,000 deaths each year in the United States (1). C. difficile infections (CDIs) are estimated to lead to almost $1 billion in excess medical costs annually in the United States (1). C. difficile infections often occur due to disruption of the microflora caused by antibiotic treatment (2, 3). A healthy microbiota helps prevent C. difficile colonization and subsequent infection. Evidence suggests that antibiotic treatment disrupts the microbiome, which leads to changes in the production of bile salts to which C. difficile is sensitive (4–9). These changes are correlated with resistance to C. difficile infection (8, 10). However, competition for nutrients may also play an important role. C. difficile can utilize amino acids as a sole carbon and energy source by using Stickland metabolism where one amino acid is an electron donor, while another amino acid is an electron acceptor (11, 12). Recent evidence has found a correlation with nutrient limitation, caused by species in the microbiota that perform Stickland metabolism, may be a factor involved in microbiota-mediated inhibition of C. difficile colonization (13). Treatment of C. difficile infections often requires antibiotic treatments, which can lead to further disruption of the microbiome and can predispose individuals to recurrent C. difficile infections (14–16).

Surotomycin, a daptomycin derivative, was developed as a treatment option for C. difficile, and its efficacy to treat C. difficile infections was the subject of phase III clinical trials. The results of trials led to conflicting interpretations, one stating that surotomycin displayed equivalent or better efficacy in treating a CDI compared to vancomycin and the other stating that surotomycin did not display any improvement over vancomycin (17–19). Daptomycin is a cyclic lipopeptide that is used to treat antibiotic-resistant strains of Enterococcus faecalis and Staphylococcus aureus (20–23). Daptomycin was long thought to exert antimicrobial activity by depolarization of the membrane (24, 25); however, recent evidence suggests the mechanism may be more complicated. Daptomycin can form a complex with the peptidoglycan biosynthesis intermediates lipid II, UPP, and UP in the presence of phosphatidylglycerol (PG), and this complex is thought to inhibit PG biosynthesis (26, 27). Daptomycin also activates the LiaRS two-component regulatory system in Bacillus subtilis, which is activated by other lipid II binding antibiotics (28–30). Since daptomycin affects components of both the cell wall and lipid membrane, it can serve as a useful tool to dissect cell envelope biogenesis.

The lipid membranes of bacteria are an essential component of the cell envelope. The lipid membrane acts as an additional barrier to the environment, maintains the proton motive force, is utilized for synthesis of ATP, and sequesters nutrients. Since the membrane is integral to a myriad of cellular processes, proper synthesis and maintenance of the membrane are critical for survival. While membrane lipid synthesis has been studied in model organisms such as Escherichia coli and B. subtilis, it is unclear if these findings translate to less studied organisms such as C. difficile. E. coli generally has a membrane composition of ~70% phosphatidylethanolamine (PE), 25% phosphatidylglycerol, and 5% cardiolipin (31, 32). B. subtilis has a membrane composition of ~70% phosphatidylglycerol, ~12% PE, ~5% cardiolipin, ~2% lysophosphatidylglycerol, and ~8% glycolipids (33).

The polar membrane lipids of C. difficile have not been well studied, and the polar lipid composition was only recently determined (34). The C. difficile membrane contains ~30% phosphatidylglycerol, 16% cardiolipin, and 50% glycolipids (34). Notably, among the phospholipids in C. difficile, no PE, phosphatidylserine (PS), or lysophosphatidylglycerol has been identified (34). There are several glycolipids that make up the lipidome of the C. difficile membrane, including monohexose-diradylglycerol (MHDRG) (14%), dihexose-diradylglycerol (DHDRG) (15%), trihexose-diradylglycerol (THDRG) (5%), and a novel glycolipid, aminohexosyl-hexosyl-diradylglycerol (HNHDRG) (16%) (34). In C. difficile, the phospholipids cardiolipin and phosphatidylglycerol contained significant amounts of plasmalogens (34). Plasmalogen species are lipids where one of the ester bonds has been substituted with an ether (35, 36); thus, we refer to the lipids as diradylglycerol rather than diacylglycerol. The specific sugar(s) on these glycolipids is not known; thus we refer to them as hexosyl-diradylglycerol. While MHDRG and DHDRG have been identified in other bacteria (33, 37, 38), it is important to note that HNHDRG has, to date, only been identified in C. difficile (34).

Two-component systems (TCSs) are a large family of regulatory systems that can be found in many forms of life, including many prokaryotes and some eukaryotes, particularly yeasts and plants (39). The function of TCSs can be quite varied, and in the case of many pathogens, TCSs often regulate virulence factors and drug resistance mechanisms (40–45). C. difficile is predicted to have approximately 50 TCSs, with slight variations being observed between strains (46). However, the vast majority of these TCSs remain unstudied, with only a few TCSs having any identified function or signal. The *cprABCK* locus has been found to respond to a subset of lantibiotic compounds and regulate the expression of antibiotic resistance mechanisms, mainly in the form of an ABC transporter (47, 48). This system shares a similar mechanism to the bacitracin resistance TCS in B. subtilis, *bceRSAB* (49, 50). The response regulator Spo0A is a major regulator of sporulation; however, its cognate histidine kinase has not been identified, and the manner in which Spo0A becomes activated is still unclear (51–53). The WalRK TCS is essential and required for proper cell wall morphology in C. difficile (54). The genes encoding the CmrRST TCS are phase variable and consist of two response regulators (RRs) and a single histidine kinase (HK). CmrRST regulates a variety of cell functions, including colony morphology and motility (55, 56). The *agrACDB* locus, homologous to the *agr* locus in S. aureus, modulates virulence by increasing production of flagella and TcdA (57–59). The RR CD1688 has been shown to be a repressor of sporulation that functions by inhibiting expression of *spoIIR*, an important early-stage regulator of sporulation (60). Loss of CD1688 leads to increased sporulation (60). Understanding the roles of these unstudied TCSs can give a better understanding of how C. difficile responds to its environment, particularly the complex host environment.

Here, we describe the identification of the TCS, *hexRK*, and its regulon, *hexSDF*. We show that HexRK and HexSDF mediate daptomycin and bacitracin resistance in C. difficile. We also show that HexSDF is required for synthesis of the novel glycolipid HNHDRG and that loss of HexSDF leads to large changes in lipid content of the cell membrane.

## RESULTS

### Identification of *hexRK* and *hexSDF*.

We performed a transposon insertion sequencing (Tn-seq) experiment in which we sought to identify genes that are required for growth in the presence of daptomycin. We generated a transposon insertion library containing ~80,000 colonies using pRPF215 as previously described (61). The transposon library was then exposed to 0 and 0.5 μg/mL daptomycin. Upon sequencing, we found there were ~45,000 unique transposon insertions, which was lower than expected. However, we identified one gene of interest, *cdr20291_2610* (here referred to as *hexK* for histidine kinase regulating synthesis of a hexose-based glycolipid), which had fewer insertions recovered when cells were grown with daptomycin (see Table S1 in the supplemental material). *hexK* is predicted to encode a TCS sensor histidine kinase (HK) with 2 transmembrane domains and an approximately 120-amino-acid extracytoplasmic domain as predicted by DeepTMHMM (62). *hexK* is immediately downstream of *cdr20291_2611* (here referred to as *hexR* for response regulator controlling synthesis of a hexose-based glycolipid), a predicted TCS RR. Through bioinformatic analysis, we found that HexK is homologous to the relatively uncharacterized HK from B. subtilis, YrkQ (Fig. S1). Due to our interest in signal transduction, we focused on HexRK for survival in the presence of daptomycin.

10.1128/mbio.03397-22.1 FIG S1 Protein alignment of HexK from C. difficile R20291 and YrkQ from B. subtilis 168 made using ClustalW. The homology between HexK and YrkQ was identified through BlastP (73). ClustalW distinguishes identical residues (*), strongly similar residues (:), and weakly similar residues (.). Download FIG S1, TIF file, 0.8 MB. Copyright © 2023 Pannullo et al. 2023 Pannullo et al. https://creativecommons.org/licenses/by/4.0/ This content is distributed under the terms of the Creative Commons Attribution 4.0 International license.

10.1128/mbio.03397-22.7 TABLE S1 Tn-seq statistics of the hex cluster. Download Table S1, PDF file, 0.1 MB. Copyright © 2023 Pannullo et al. 2023 Pannullo et al. https://creativecommons.org/licenses/by/4.0/ This content is distributed under the terms of the Creative Commons Attribution 4.0 International license.

**FIG 1:**
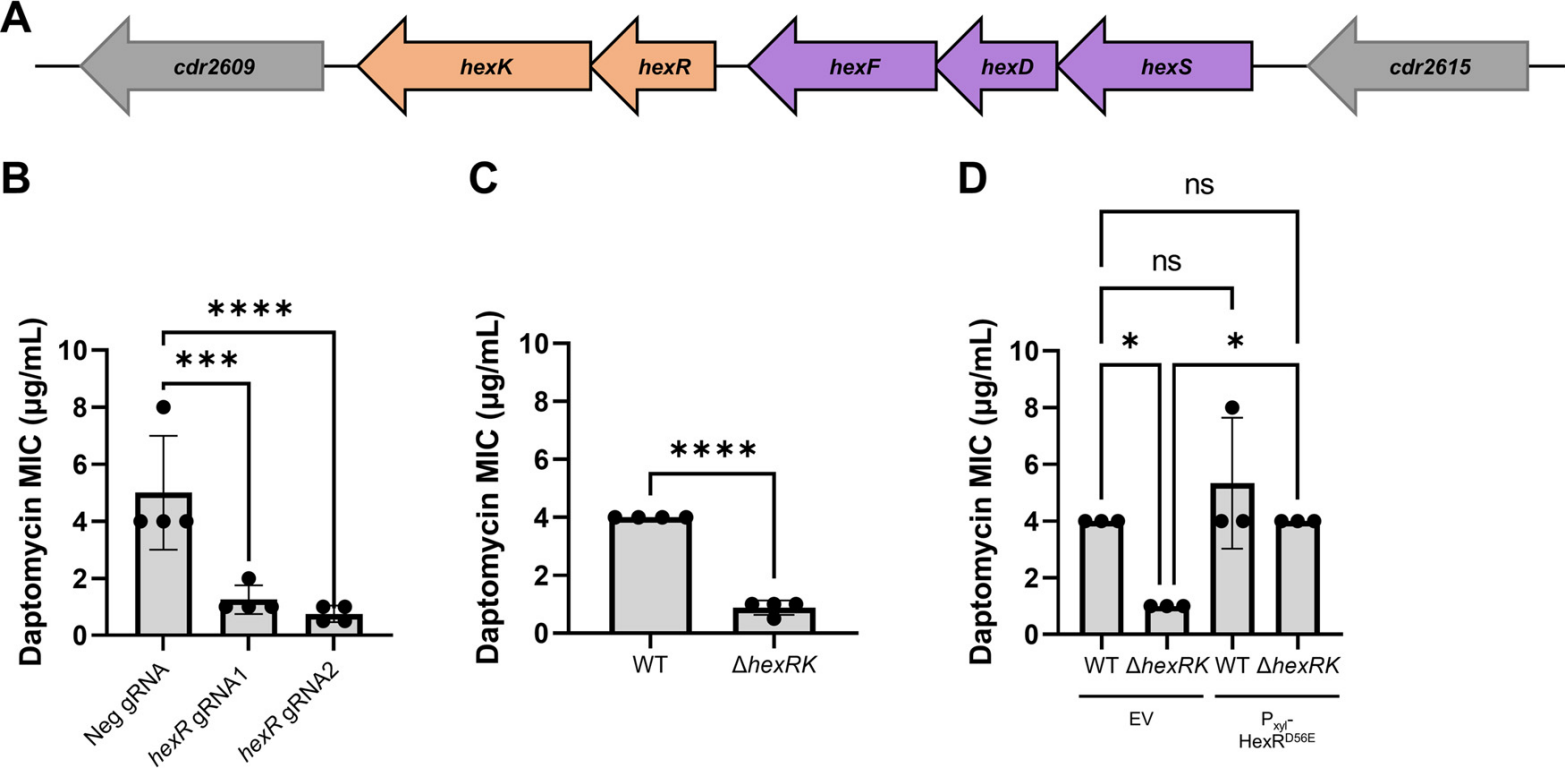
*hexRK* and *hexSDF*. (A) Diagram of the *hex* cluster and neighboring genes. *hexK* (*cdr2610*) is annotated as a TCS histidine kinase, and *hexR* (*cdr2611*) is annotated as a TCS response regulator. *hexF* (*cdr2612)* is annotated as a MprF-like flippase, *hexD* (*cdr2613*) is annotated as a polysaccharide deacetylase, and *hexS* (*cdr2614*) is annotated as a monogalactosyldiacylglycerol synthase. The neighboring gene adjacent to *hexK* is *cdr2609* and is annotated as a putative membrane protein. The gene neighboring *hexS* is *cdr2615* (*glyA*) and is annotated as a putative serine hydroxymethyltransferase. (B) MICs of CRISPRi knockdown with 2 guides, each targeting a different location within *hexR.* These knockdowns result in ~4-fold decreases in daptomycin resistance. Data were analyzed using a one-way analysis of variance (ANOVA) with Dunnett’s multiple-comparison test. ****, *P* < 0.0001; ***, *P* < 0.001. (C) Δ*hexRK* has ~4-fold-lower daptomycin MIC than WT. Data were analyzed with an unpaired *t* test; ****, *P < *0.0001. (D) Overproduction of HexR^D56E^ in Δ*hexRK* restores daptomycin resistance to WT levels compared to Δ*hexRK* with empty pAP114 (EV). Data were analyzed using one-way ANOVA using Sidak’s multiple-comparison test. *, *P* < 0.05; ns, *P* > 0.05.

To confirm that loss of *hexRK* led to a decrease in daptomycin resistance, as the Tn-seq data suggested, we utilized CRISPR interference (CRISPRi) to knock down expression of the *hexRK* operon. We found knockdown of *hexR* leads to an ~4-fold decrease in daptomycin resistance (Fig. 1B). We then constructed a Δ*hexRK* deletion using CRISPR mutagenesis, and we found that loss of HexRK decreased daptomycin resistance ~4-fold (Fig. 1C). We then asked if we could restore daptomycin resistance by expression of HexR^D56E^ (a mutant of the putative phosphorylation site), which mimics phosphorylation of HexR and likely increases HexR activity. When *hexR^D56E^* is expressed from a xylose-inducible promoter in a *ΔhexRK* mutant, we observed an increase in daptomycin resistance to near-wild-type levels (Fig. 1D). Taken together, our data suggest that HexRK is required for daptomycin resistance.

### Identification of the HexR regulon.

We hypothesized that HexRK likely regulates expression of genes involved in daptomycin resistance. Thus, we performed transcriptome sequencing (RNA-seq), comparing WT with Δ*hexRK* to identify any genes that were differentially regulated in the absence of *hexRK*. Previous RNA-seq experiments showed *hexRK* was expressed at relatively high levels in log-phase growth (54); thus, we performed RNA-seq experiments on cultures grown to mid-log phase (optical density at 600 nm [OD_600_], 0.7 to 0.8) (Table S2).

10.1128/mbio.03397-22.8 TABLE S2 WT versus ΔhexRK (expression of ΔhexRK/WT) RNA-seq full dataset. Download Table S2, XLSX file, 0.2 MB. Copyright © 2023 Pannullo et al. 2023 Pannullo et al. https://creativecommons.org/licenses/by/4.0/ This content is distributed under the terms of the Creative Commons Attribution 4.0 International license.

**FIG 2:**
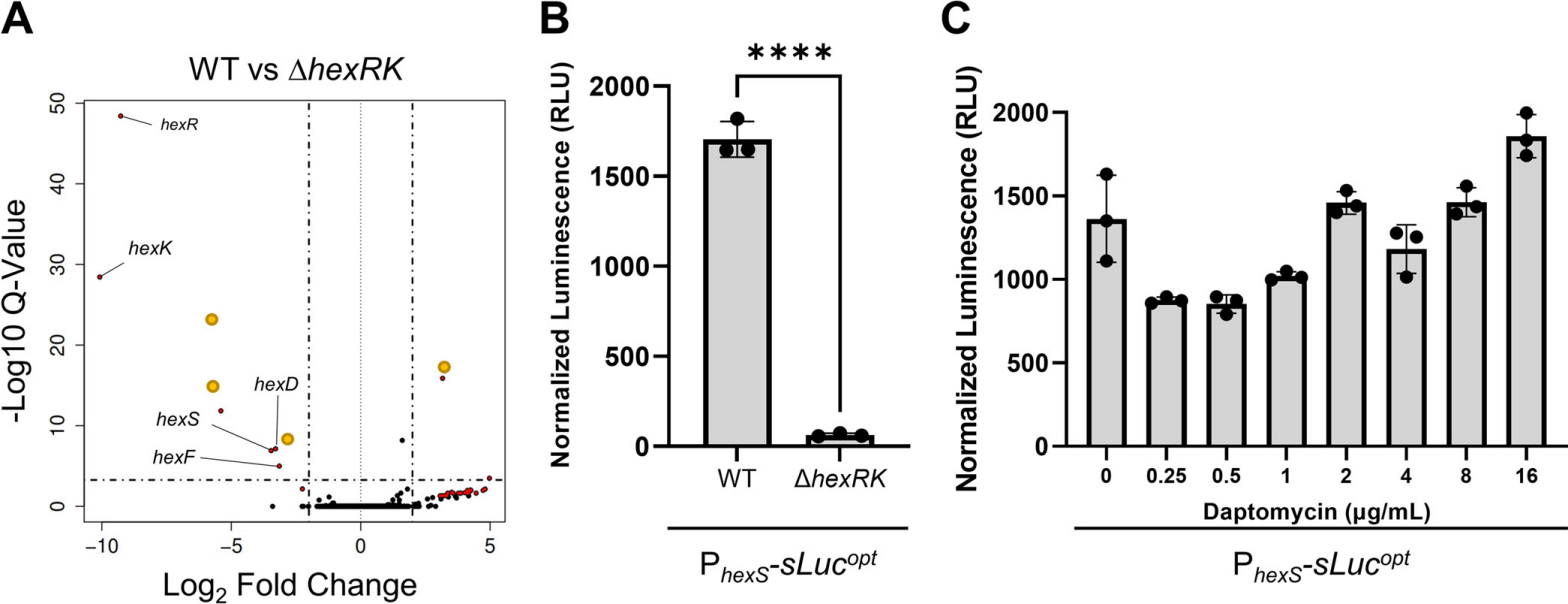
Expression of *hexSDF* is regulated by HexRK. (A) Volcano plot of RNA-seq data comparing WT and Δ*hexRK* shows a decrease in the expression *hexSDF* and relatively few other changes in the transcriptome. Genes that have been previously reported to be phase variable have been highlighted with a yellow dot. Genes neighboring the *hex* cluster did not show altered expression in Δ*hexRK* (see Table S2 in the supplemental material). (B) The luciferase reporter P*_hexS_*-*sLuc^opt^* shows significantly decreased activity in Δ*hexRK* compared to WT, confirming the differential expression identified in RNA-seq. Data were analyzed with an unpaired *t* test; ****, *P > *0.0001. (C) The P*_hexS_*-*sLuc^opt^* reporter does not show altered luminescence in the presence of multiple concentrations of daptomycin after incubation for 2 h.

We found that 32 genes, not including *hexRK*, were differentially regulated between wild type (WT) and Δ*hexRK* with a *P* value of <0.05 and a log_2_ fold change of greater than 2 or less than −2. Of these 32 genes in WT versus Δ*hexRK*, 24 showed an increase in expression, and 8 showed a decrease in expression (Table 1; Fig. 2A). Since our data suggest that HexRK is required for daptomycin resistance, we were most interested in those genes whose expression was dependent upon HexRK. We were particularly interested in a three-gene operon, *cdr20291_2614-2613-2612* (*hexSDF*), located immediately upstream of *hexRK* (Fig. 1A). We have named the genes *hexSDF* due to their involvement in the synthesis of a hexose-based glycolipid, and based on predictions, HexS appears as a synthase, HexD a deacetylase, and HexF a flippase. The genes in the *hexSDF* operon were expressed ~12-fold lower in the Δ*hexRK* mutant (Table 1; Fig. 2A). The RNA-seq revealed other differentially regulated genes; however, several of these genes are reported to be phase variable, including *cdr0440*, *cdr0962*, *cdr0963*, and *cdr1514* (63–66). As such, we chose to focus our efforts on better understanding the function of the *hexSDF* operon. To confirm that HexRK is required for *hexSDF* expression, we constructed a P*_hexS_-sLuc^opt^* reporter. We found that expression of the P*_hexS_-sLuc^opt^* reporter was reduced by 26-fold in Δ*hexRK* compared to WT, indicating that expression of *hexSDF* is dependent upon HexRK (Fig. 2B). Since we found that daptomycin resistance decreases in the absence of HexRK, we were interested if daptomycin was an activator of the HexRK system. We tested if the P*_hexS_-sLuc^opt^* was induced after a 2-h treatment with various concentrations of daptomycin. We found, under these conditions, that daptomycin did not lead to increased expression of the *hexSDF* locus (Fig. 2C). We also tested growth with antibiotics for up to 6 h but did not detect any induction.

**TABLE 1:** WT versus Δ*hexRK* (expression of Δ*hexRK*/WT) RNA-seq genes of interest

Gene ID	Log_2_ fold change	*P* value (BH FDR corrected)^*a*^	Gene annotation
CDR20291_2611	−10.08210496	2.03E-32	Two-component response regulator
CDR20291_2610	−9.273771535	1.09E-52	Two-component sensor histidine kinase
CDR20291_0963	−5.709317004	2.65E-18	Putative uncharacterized protein
CDR20291_0962	−5.701107822	5.23E-27	Putative uncharacterized protein
CDR20291_1078	−5.402045749	2.80E-15	Hypothetical protein
CDR20291_2614	−3.459697452	3.80E-10	Putative UDP-*N*-acetylglucosamine-*N*-acetylmuramyl–(pentapeptide) pyrophosphoryl-undecaprenol *N*-acetylglucosamine transferase
CDR20291_2613	−3.288677169	2.05E-10	Probable polysaccharide deacetylase
CDR20291_2612	−3.144832994	3.45E-08	Putative membrane protein
CDR20291_1514	−2.799807865	1.65E-11	Putative signaling protein
CDR20291_2123	−2.253107726	2.71E-05	Hypothetical protein
CDR20291_3418	3.069822103	0.000532045	Conserved hypothetical protein
CDR20291_0921	3.16028253	0.00045218	Hypothetical protein
CDR20291_2121	3.163664373	1.85E-19	Putative regulatory protein
CDR20291_2122	3.233236824	1.72E-20	Putative regulatory protein
CDR20291_3482	3.26608685	0.000470265	Hypothetical protein
CDR20291_0521	3.30548934	0.000473497	Hypothetical protein
CDR20291_1811	3.32727842	0.000427186	ABC transporter substrate binding protein
CDR20291_0922	3.362368022	0.000174603	Hypothetical protein
CDR20291_3193	3.512740019	0.000113211	Putative exosporium glycoprotein
CDR20291_0522	3.572642397	0.000205762	Putative spore coat protein
CDR20291_2867	3.774265869	0.00020729	Putative aminotransferase
CDR20291_1511	3.85797267	0.000207838	Hypothetical protein
CDR20291_0523	3.865297775	0.000201116	Putative spore coat protein
CDR20291_1741	4.021590909	0.000133238	Putative membrane protein precursor
CDR20291_1740	4.08897198	9.57E-05	Putative RNA methyltransferase;
CDR20291_3194	4.132460956	5.80E-05	Putative glycosyl transferase
CDR20291_1282	4.14416368	0.000178136	Putative bifunctional protein, peroxiredoxin/chitinase
CDR20291_1825	4.170182329	0.00014694	ABC transporter ATP binding protein
CDR20291_0476	4.238098841	4.55E-05	Putative spore cortex-lytic enzyme pre-pro form (putative spore peptidoglycan hydrolase)
CDR20291_0926	4.260110287	5.80E-05	Hypothetical protein
CDR20291_2291	4.460472396	0.000181495	Putative spore coat protein
CDR20291_2289	4.729664887	5.47E-05	Hypothetical protein
CDR20291_2290	4.808401936	3.24E-05	Putative spore coat protein
CDR20291_0440	4.974475819	1.20E-06	Cell surface protein (putative hemagglutinin/adhesin) precursor

a BH FDR, Benjamini-Hochberg false-discovery rate.

### Loss of *hexRK* and *hexSDF* leads to daptomycin and bacitracin susceptibility.

HexS is annotated as a putative monogalactosyldiacylglycerol synthase, HexD is a predicted polysaccharide deacetylase, and HexF is annotated as MprF-like. In other organisms, MprF is known to contribute to daptomycin resistance (Fig. 1A) (67–69). MprF from S. aureus and B. subtilis is a bifunctional protein that adds lysine onto phosphatidylglycerol, generating Lys-PG, and then flips Lys-PG from the inner leaflet of the membrane to the outer leaflet (70–72). Bioinformatic analysis reveals that HexF contains homology to the flippase domain of MprF, but HexF does not appear to contain the domain required for lipid lysinylation (Fig. S2). Based on the bioinformatic predictions of the *hexSDF* operon, we hypothesized that the operon likely encodes proteins that alter biosynthesis of membrane lipids.

10.1128/mbio.03397-22.2 FIG S2 Protein alignment of HexF from C. difficile R20291 and B. subtilis 168 MprF (BSU08425, C0H3X7). Alignment was made using ClustalW. The flippase domain of B. subtilis MprF has been highlighted in red. ClustalW distinguishes identical residues (*), strongly similar residues (:), and weakly similar residues (.). Download FIG S2, TIF file, 0.8 MB. Copyright © 2023 Pannullo et al. 2023 Pannullo et al. https://creativecommons.org/licenses/by/4.0/ This content is distributed under the terms of the Creative Commons Attribution 4.0 International license.

**FIG 3:**
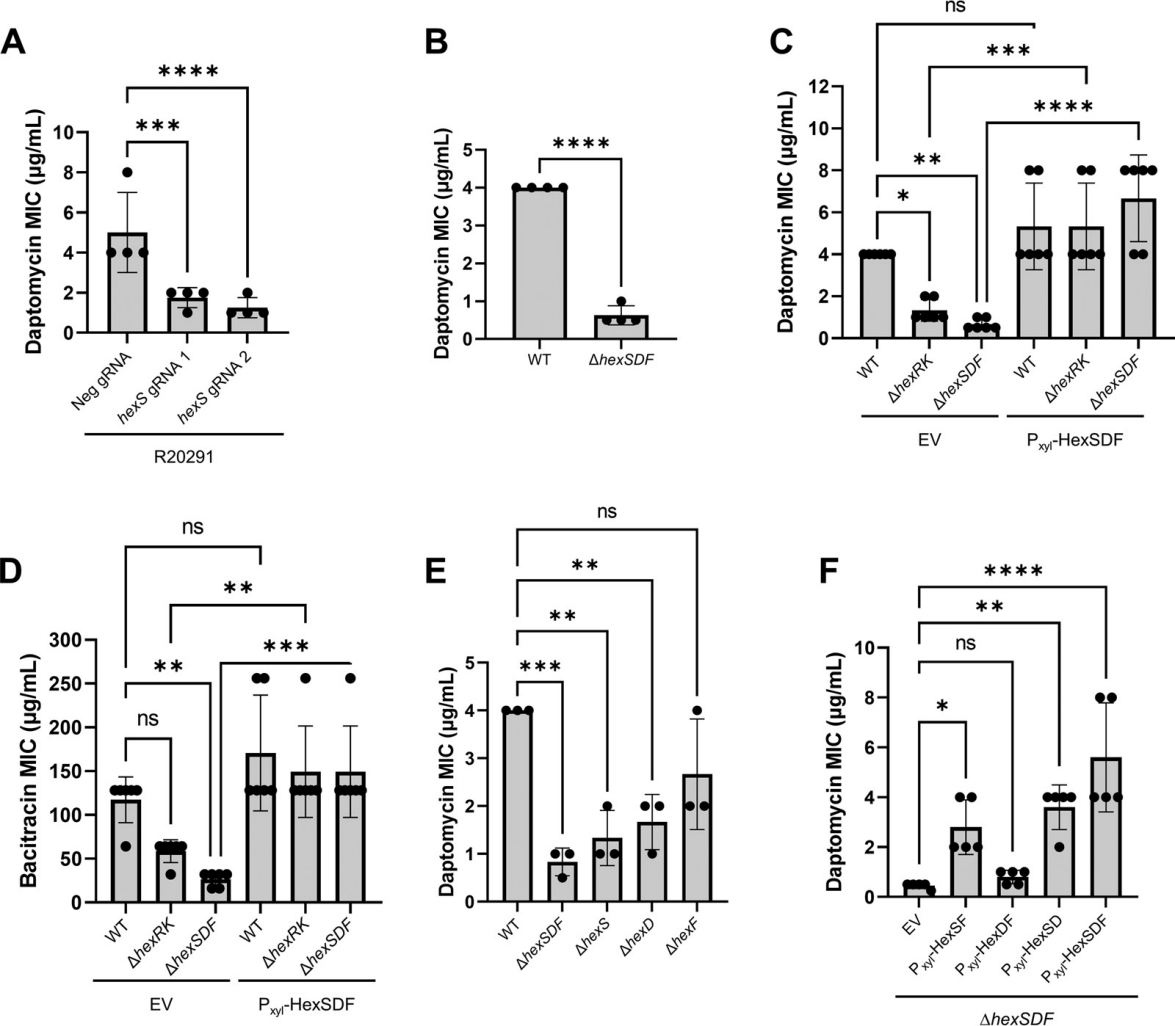
HexRK and HexSDF are involved in daptomycin and bacitracin resistance. (A) CRISPRi knockdown of *hexS* leads to an ~4-fold decrease in daptomycin resistance. (B) Deletion of *hexSDF* results in an ~8-fold decrease in daptomycin MIC. Data were analyzed using unpaired *t* test; ****, *P < *0.0001. (C) Overproduction of HexSDF restores daptomycin resistance in Δ*hexSDF*. (D) Deletion of *hexSDF* results in an ~4-fold decrease in bacitracin MIC, and overproduction of HexSDF restores bacitracin resistance in Δ*hexSDF*. Data in panels C and D were analyzed using one-way ANOVA with Sidak’s multiple-comparison test. ****, *P* < 0.0001; ***, *P* < 0.001; **, *P* < 0.01; *, *P* < 0.05; ns, *P* > 0.05. (E) Deletion of *hexS* or *hexD* decreases daptomycin resistance. (F) Overproduction of HexSD, HexSF, and HexSDF leads to increases in daptomycin MIC in Δ*hexSDF*. Unless otherwise specified, data were analyzed using one-way analysis of variance using Dunnett’s multiple-comparison test. ****, *P* < 0.0001; ***, *P* < 0.001; **, *P* < 0.01; ns, *P* > 0.05.

To determine if HexSDF is required for daptomycin resistance, we utilized CRISPRi to knock down expression of the *hexSDF* operon. We found that knockdown of *hexSDF* led to a ~4-fold decrease in daptomycin MIC (Fig. 3A). Having demonstrated that CRISPRi knockdowns of *hexSDF* decreased daptomycin resistance, we constructed a deletion of the *hexSDF* operon (Δ*hexSDF*) and found that this resulted in an ~8-fold decrease in daptomycin resistance compared to WT (Fig. 3B). We then complemented these mutants by expressing *hexSDF* from a xylose-inducible promoter. We found that overproduction of HexSDF in WT C. difficile R20291 did not lead to significant increases in daptomycin resistance; however, when HexSDF is overproduced in either *ΔhexRK* or Δ*hexSDF*, daptomycin resistance is restored to WT levels (Fig. 3C). Taken together, these data suggest that HexSDF is required for HexRK-mediated daptomycin resistance.

We sought to determine if loss of *hexSDF* affected resistance to other antimicrobials. We screened several other cell wall-targeting antimicrobials, including vancomycin, bacitracin, nisin, polymyxin, ampicillin, and lysozyme. We found that loss of *hexSDF* decreased bacitracin resistance by ~4-fold (Fig. 3D) but did not result in altered resistance to other antimicrobials (Fig. S3). Expression of *hexSDF* in Δ*hexSDF* resulted in bacitracin MIC comparable to WT; however, overproduction of HexSDF in WT did not affect bacitracin MIC (Fig. 3D).

10.1128/mbio.03397-22.3 FIG S3 Δ*hexSDF* MICs of other antimicrobials including vancomycin (A), nisin (B), polymyxin B (C), ampicillin (D), and lysozyme (E). Data were analyzed using an unpaired *t* test. ns, *P* > 0.05. Download FIG S3, TIF file, 0.7 MB. Copyright © 2023 Pannullo et al. 2023 Pannullo et al. https://creativecommons.org/licenses/by/4.0/ This content is distributed under the terms of the Creative Commons Attribution 4.0 International license.

We wanted to better understand the contribution of each gene in the *hexSDF* operon to daptomycin and bacitracin resistance. Thus, we constructed deletions of each gene individually. We found that loss of *hexS* had the greatest effect on daptomycin resistance, decreasing the daptomycin MIC by ~4-fold (Fig. 3E). The *hexD* and *hexF* mutations lead to an ~2-fold decrease in the daptomycin resistance (Fig. 3E). We also investigated how the individual deletions affected bacitracin resistance and found trends similar to what was found with daptomycin (Fig. S4A). We next overproduced pairs of genes from the *hex* operon and found that P_xyl_-*hexSF*, P_xyl_-*hexSD*, and P_xyl_-*hexSDF* all led to increases in daptomycin resistance (Fig. 3F), while P_xyl_-*hexDF* did not lead to an increase in resistance (Fig. 3F). We found that the bacitracin MICs showed a remarkably similar trend where HexS expression is essential for increased bacitracin resistance (Fig. S4B). Combined, these data indicate that HexS is essential for increased daptomycin and bacitracin resistance.

10.1128/mbio.03397-22.4 FIG S4 The *hex* locus mediates resistance to bacitracin. (A) Δ*hexS* and Δ*hexD* led to significant decreases in bacitracin MIC compared to WT; however, Δ*hexF* did not have a significant decrease in bacitracin MIC. (B) Bacitracin MIC comparing overproduction of HexSF, HexSD, and HexDF in Δ*hexSDF*. Overproduction of HexSF and HexSD led to mild increases in bacitracin resistance, while overproduction of HexDF did not lead to notable increases in bacitracin resistance. (C) P*_hexS_*-*sLuc^opt^* did not show altered luminescence when exposed to varying concentrations of bacitracin for 2 hours. Data were compared by one-way ANOVA. ***, *P* < 0.001; **, *P* < 0.01; ns, *P* > 0.05. Download FIG S4, TIF file, 0.6 MB. Copyright © 2023 Pannullo et al. 2023 Pannullo et al. https://creativecommons.org/licenses/by/4.0/ This content is distributed under the terms of the Creative Commons Attribution 4.0 International license.

Finally, we wanted to determine if HexSDF is important for daptomycin resistance in other strains of C. difficile. C. difficile 630 (PCR ribotype 012) is a widely used and well-studied strain of C. difficile and thus the most appropriate strain to use for comparison to our findings in R20291. Strain 630, like nearly all strains of C. difficile, encodes the *hex* locus. We utilized CRISPRi to knockdown *hexSDF* in 630Δ*erm* and found, similar to R20291, an ~2- to 4-fold decrease in daptomycin MIC compared to the Neg sgRNA (Fig. S5). This suggests that HexSDF is involved in daptomycin resistance in multiple strains of C. difficile.

10.1128/mbio.03397-22.5 FIG S5 CRISPRi knockdown of *hexS* in 630Δ*erm* leads to a decrease in daptomycin MIC. Data were analyzed with one-way analysis of variance using Dunnett’s multiple-comparison test. ***, *P* < 0.001; **, *P* < 0.01. Download FIG S5, TIF file, 0.3 MB. Copyright © 2023 Pannullo et al. 2023 Pannullo et al. https://creativecommons.org/licenses/by/4.0/ This content is distributed under the terms of the Creative Commons Attribution 4.0 International license.

### HexSDF is required for production of HNHDRG.

Based on bioinformatic predictions of the *hexSDF* operon, we hypothesized that the alterations in daptomycin and bacitracin resistance were due to changes in membrane lipid composition. Several lines of evidence supported this hypothesis. First, HexS is annotated as a monogalactosyldiacylglycerol synthase, indicating that it likely is adding sugar groups to a lipid (46, 73). Second, HexF is annotated as an MprF-like protein. In other organisms such as S. aureus, MprF is known to contribute to daptomycin resistance by converting phosphatidylglycerol into lysophosphatidylglycerol (67–69). Importantly, HexF only contains homology to the flippase domain of MprF (Fig. S2), meaning that HexF could play a role in flipping lipids across the membrane. Finally, HexD is annotated as a putative polysaccharide deacetylase, potentially removing an *N*-acetyl group, resulting in the production of an amino sugar. Previous work defining the polar lipids in C. difficile revealed the presence of a glycolipid with an amino sugar HNHDRG (34). Based on these bioinformatic predictions, we hypothesized that HexSDF is required for production of the novel glycolipid HNHDRG.

**FIG 4:**
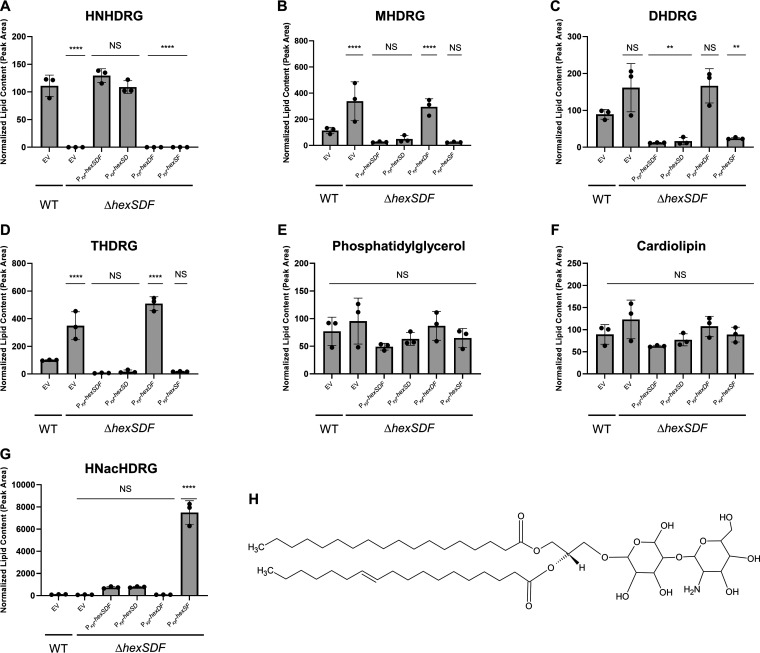
HexSDF is required for synthesis of HNHDRG. Compared to WT, Δ*hexSDF*, Δ*hexSDF* P_xyl_-*hexDF*, and Δ*hexSDF* P_xyl_-*hexSF* show significantly decreased levels of HNHDRG (A) and increased amounts of MHDRG (B), DHDRG (C), and THDRG (D). None of the tested strains had significant differences in phosphatidylglycerol (E) or cardiolipin (F) content. (G) Interestingly, Δ*hexSDF* P_xyl_-*hexSF* leads to the accumulation of the HNHDRG acetylated intermediate, termed HNacHDRG. (H) Structure of HNHDRG. Data were analyzed with one-way analysis of variance using Dunnett’s multiple-comparison test. ****, *P* < 0.0001; **, *P* < 0.01; ns, *P* > 0.05.

To determine if HexSDF was required for production of glycolipids, we performed an untargeted lipidomics experiment comparing WT empty vector (EV), *ΔhexSDF* EV, *ΔhexSDF* P*_xyl_-hexSDF*, *ΔhexSDF* P*_xyl_*-*hexSD*, *ΔhexSDF* P*_xyl_*-*hexSF*, and *ΔhexSDF* P*_xyl_*-*hexDF*. Overnight cultures were subcultured in tryptone-yeast (TY) supplemented with 1% xylose to an OD_600_ of 0.05 and were grown to an OD_600_ of 0.6 to 0.7, at which point the cells were harvested. The membrane lipids were extracted and analyzed by high-performance liquid chromatography–tandem mass spectrometry (HPLC-MS/MS) to identify and quantify lipid species. The most striking difference we observed was the complete loss of the unique C. difficile glycolipid HNHDRG in the Δ*hexSDF* mutant compared to WT (Fig. 4A). When HexSDF is overproduced in a Δ*hexSDF* mutant, HNHDRG is detected in similar levels to WT (Fig. 4A). Interestingly, we found that the three other major glycolipids, MHDRG (Fig. 4B), DHDRG (Fig. 4C), and THDRG (Fig. 4D), increased in Δ*hexSDF* compared to WT. The levels of all three glycolipids were also decreased to levels lower than WT when HexSDF was overproduced (Fig. 4B to D). In contrast, phosphatidylglycerol (Fig. 4E) and cardiolipin (Fig. 4F) levels were not significantly different in the Δ*hexSDF* mutant or strains overproducing HexSDF; however, the levels of both did trend upward in the *ΔhexSDF* mutant.

We next sought to determine the contribution of individual genes in the *hex* operon. We found overproduction of HexDF did not lead to accumulation of either HNHDRG (Fig. 4A). This suggests that HexS is absolutely required for HNHDRG production. Interestingly, we found that overproduction of HexSF leads to accumulation of HNacHDRG, a previously undetected glycolipid which we predict is the acetylated intermediate of HNHDRG, but there was no detectable HNHDRG (Fig. 4A and G). This suggests that the putative deacetylase HexD is required for deacetylation of HNacHDRG to produce HNHDRG. In contrast, overproduction of HexSD leads to accumulation of near-wild-type levels of HNHDRG (Fig. 4G), suggesting HexF is not essential for HNHDRG production. *In toto*, we hypothesize that HexS is required for incorporation of *N*-acetyl-hexosyl onto the MHDRG precursor, resulting in an HNacHDRG intermediate, and that HexD is required for deacetylation, which leads to production of HNHDRG.

## DISCUSSION

### Identification of an operon required for production of a novel glycolipid.

The C. difficile membrane consists of ~50% glycolipids, including MHDRG, DHDRG, THDRG, and HNHDRG. We do not currently know the nature of the hexosyl groups on these lipids, and in fact, we do not know if MHDRG is a single type of glycolipid or a mixture of two or more glycolipids with different sugars. HNHDRG is a novel glycolipid that makes up ~16% of the membrane WT C. difficile and, to date, has not been described in other bacteria (34). The principal contribution of this investigation is the identification of HexRK and HexSDF as essential for the production of the novel glycolipid HNHDRG. We identified these genes in a Tn-seq screen for genes involved in daptomycin resistance. We found that knockdowns or knockouts of either *hexRK* or *hexSDF* lead to decreases in daptomycin and bacitracin resistance. We find that in the absence of HexSDF, there is almost no detectable HNHDRG. We could complement these defects with a plasmid expressing *hexSDF* from a xylose-inducible promoter. This argues that HexSDF is required for resistance to daptomycin and bacitracin as well as production of the novel glycolipid HNHDRG.

### Model for HNHDRG synthesis.

**FIG 5:**
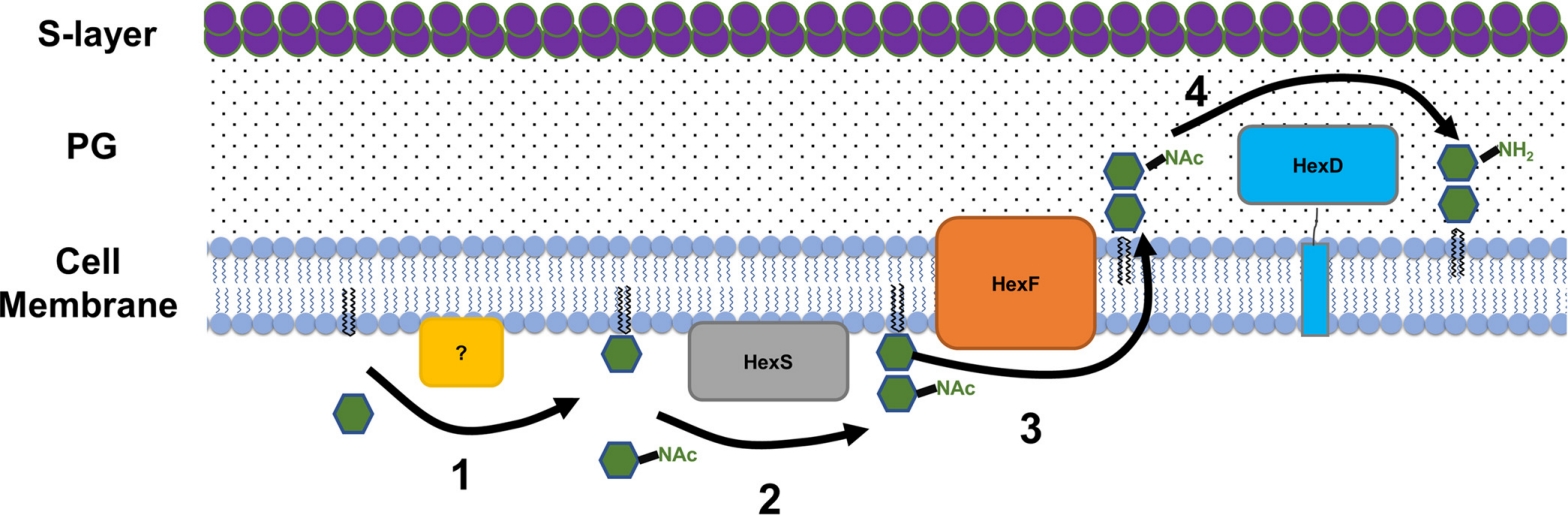
HexRK and HexSDF model. (1) We hypothesize that the first step in HNHDRG synthesis is production of MHDRG; the gene(s) required for MHDRG synthesis is not known. (2) In the next proposed step, HexS utilizes MHDRG as its substrate and adds an *N*-acetyl-hexose, forming the HNacHDRG intermediate. (3) HNacHDRG is then flipped outside the cell via HexF (and potentially other flippases). (4) Once on the external side of the membrane, NHacHDRG is deacetylated by HexD to form HNHDRG. Localization of HexSDF has not been experimentally determined; this model represents the proposed localization of each protein based on bioinformatic predictions.

Based on the lipidomics data, we hypothesize HNHDRG is likely produced by HexS catalyzing the addition of an *N*-acetyl-hexose to an MHDRG (Fig. 5). This lipid is then flipped to the outer leaflet of the membrane by HexF, and the *N*-acetyl group is deacetylated by HexD, producing HNHDRG on the outer leaflet of the membrane (Fig. 5). This model suggests deacetylation is required for production of HNHDRG and is supported by lipidomics data from strains lacking HexD, which show the accumulation of HNacHDRG (Fig. 4G). While we have identified the genes required for synthesis of HNHDRG, the steps of HNHDRG synthesis are still not entirely defined. Currently, little is known about membrane lipid synthesis in C. difficile and if it differs from better-studied organisms.

In B. subtilis, all known glycolipid synthesis is dependent upon a single enzyme, UgtP, which catalyzes the addition of glucose to diacylglycerol to create monoglucosyl diacylglycerol (MGDG) (74). In B. subtilis, UgtP works processively to add an additional glucose group to MGDG to synthesize diglucosyl diacylglycerol (DGDG) and a third glucose to DGDG to create triglucosyl diacylglycerol TGDG (74). We think it is unlikely that HexS is responsible for catalyzing the addition of two different sugars, one without an *N*-acetyl group and one with an *N*-acetyl group, onto diradylglycerol. Instead, we hypothesize that another enzyme, potentially one of the three C. difficile genes predicted to encode UgtP-like homologs (*cdr20291_0008*, *cdr20291_1186*, and *cdr20291_2958*) synthesizes MHDRG and HexS and then adds *N*-acetyl-hexosyl to MHDRG to generate HNacHDRG. This is supported by our data showing that HexS is absolutely required for HNHDRG synthesis. Consistent with this model, we find increased levels of MHDRG, DHDRG, and THDRG in all strains lacking HexS. We propose that in the absence of HexS, MHDRG is no longer being consumed to generate HNHDRG; thus, more MHDRG is available to be used to synthesize DHDRG and THDRG. We also find that overproduction of HexS leads to significant decreases of MHDRG, DHDRG, and THDRG, which is consistent with this model. However, at this time, we cannot rule out the possibility that HexS adds hexosyl-*N*-acetyl-hexosyl directly to diradylglycerol (DRG).

As noted earlier, we find that overproduction of HexSF without HexD results in the production of HNacHDRG but not HNHDRG. This is consistent with the role of HexD in deacetylation of HNacHDRG to produce HNHDRG. However, we were surprised that expression of HexSD without HexF produced HNHDRG at levels equivalent to all three genes alone. We hypothesize three possible explanations for this phenomenon. One possibility is that HexD functions intracellularly prior to flipping of HNHDRG. A second possibility is that HexF is not required for flipping. The third possibility is that in the absence of HexF, other flippases are capable of mediating transport/flipping of HNacHDRG, where it is acted on extracellularly by HexD. It is interesting to note that C. difficile encodes two additional genes (*cdr20291_2010* and *cdr20291_3180*) that are predicted to encode proteins with homology to HexF/MprF and may also function as flippases (Fig. S6). Taken together, these data support a model in which HexS adds *N*-acetyl-hexosyl to MHDRG, leading to production of HNacHDRG. HNacHDRG is then deacetylated by HexD to produce HNHDRG.

10.1128/mbio.03397-22.6 FIG S6 Protein multiple-sequence alignment comparing HexF, CDR2010, and CDR3180 made using ClustalW. ClustalW distinguishes identical residues (*), strongly similar residues (:), and weakly similar residues (.). Download FIG S6, TIF file, 5.8 MB. Copyright © 2023 Pannullo et al. 2023 Pannullo et al. https://creativecommons.org/licenses/by/4.0/ This content is distributed under the terms of the Creative Commons Attribution 4.0 International license.

### Identification of the HexRK regulon.

HexRK represents a family of TCSs that have undefined functions. We found that HexK shares homology with YrkQ in B. subtilis. YrkQ is a relatively unstudied TCS, and B. subtilis is not known to produce HNHDRG, nor is it known what signals activate YrkPQ. To date, we have been unable to identify a signal to which HexRK responds; neither daptomycin (Fig. 2C) nor bacitracin (see Fig. S4C in the supplemental material) leads to activation of the P*_hexS_*-*sLuc^opt^* reporter, and our RNA-seq data suggest *hexRK* has a high basal expression (54). While the signals are not known, both the HexRK and YrkPQ regulons have been identified (75). Similar to the C. difficile HexR regulon, the YrkP regulon is small, ~8 genes, and consists mostly of proteins with predicted function involved in lipid or membrane biogenesis. This includes YkcB, a predicted C_55_-P-Glc glucosyltransferase; YkcC, a predicted lipoteichoic acid glycosyltransferase; YrkO, a membrane protein with 10 predicted transmembrane domains and DUF418 domain, which may be involved in transport; YrkN, a putative GNAT *N*-acetyltransferase; and YrkR, a 4-transmembrane domain protein with homology to phosphate starvation-inducible protein PsiE. This raises the possibility that HexRK and YrkPQ are responsible for regulating genes involved in membrane or cell envelope biogenesis in multiple organisms. Consistent with this, the YrkP regulon is activated in daptomycin-resistant mutants of B. subtilis (76). However, it is not clear how much YrkPQ contributes to daptomycin resistance in B. subtilis. It is also important to note that B. subtilis does not encode an operon homologous to *hexSDF*.

### Mechanism of HNHDRG daptomycin resistance.

Since phosphatidylglycerol is required for daptomycin to kill target cells, we initially expected that loss of HexSDF may lead to an increase in phosphatidylglycerol content and thus a decrease in daptomycin resistance. However, we found there was not a significant change in phosphatidylglycerol levels in Δ*hexSDF* (Fig. 4F). The subtle changes we observed in phosphatidylglycerol are still present in strains lacking either HexD or HexF. Consistent with this, we find that loss of HexD or HexF also leads to a decrease in daptomycin resistance (Fig. 3E). Taken together, it suggests that HNHDRG is playing a more direct role in daptomycin resistance.

We are unsure how HNHDRG increases daptomycin resistance. However, one potential clue comes from the screening of the Δ*hexSDF* mutant for sensitivity to additional antibiotics, where we find HexSDF is required for resistance to bacitracin and daptomycin but not other cell wall-targeting antibiotics. It has been hypothesized that since C. difficile does not produce PE or PS, HNHDRG serves to balance the charge of the membrane, as it can form a positively charged zwitterion (34). This could potentially explain the resistance to daptomycin and bacitracin. Both daptomycin and bacitracin require cationic metal ions to function. Daptomycin requires Ca^2+^, while bacitracin prefers Zn^2+^, though it can utilize other divalent metal ions (21, 26, 77). We hypothesize that HNHDRG decreases the ability of positively charged metal ions to access the membrane, limiting the activity of both daptomycin and bacitracin. However, there is the caveat that loss of HNHDRG does not affect the activity of cationic antimicrobial peptides, such as lysozyme (Fig. S3), which one may expect if HNHDRG functioned by alteration of membrane charge. While the exact mechanism of resistance is unclear, we hypothesize that HNHDRG is affecting resistance directly, as opposed to alterations in other membrane lipid species being responsible for resistance.

## MATERIALS AND METHODS

### Bacterial strains, media, and growth conditions.

Bacterial strains are listed in Table 2. The C. difficile strains used in this study are derivatives of R20291 (78) and 630Δ*erm* (79). C. difficile strains were grown on tryptone-yeast (TY) medium that consisted of 3% tryptone, 2% yeast extract, and 2% agar (for solid medium). TY was supplemented as needed with thiamphenicol at 10 μg/mL (Thi_10_). Conjugations were performed on solid brain heart infusion (BHI) media supplemented with yeast extract (3.65% BHI, 0.5% yeast extract, 2% agar) and plated on TY with thiamphenicol at 10 μg/mL, kanamycin at 50 μg/mL, and cefoxitin at 50 μg/mL. C. difficile strains were maintained at 37°C in an anaerobic chamber (Coy Laboratory Products) in an atmosphere of ~2 to 2.5% H_2_, 5% CO_2_, and 85% N_2_.

**TABLE 2:** Strains used in this study

Species and strain	Genotype and/or description	Source or reference
E. coli strains		
OmniMAX-2 T1R	F′ [*proAB^+^ lacI*^q^ *lacZΔ*M15 Tn*10*(Tet^r^) *Δ(ccdAB*)] *mcrA Δ*(*mrr-hsdRMS-mcrBC*) φ80(*lacZ*)*Δ*M15 *Δ*(*lacZYA-argF*)*U169 endA1 recA1 supE44 thi-1 gyrA96 relA1 tonA panD*	Invitrogen
HB101/pRK24	F^−^ *mcrB mrr hsdS20*(r_B_^−^ m_B_^−^) *recA13 leuB6 ara*^−^ *14 proA2 lacY1 galK2 xyl-5 mtl-1 rpsL20*	85
MG1655	Wild type	
B. subtilis strains		
BS49	Tn*916* donor strain, Tet^r^	80
C. difficile strains		
R20291	Wild-type strain from UK outbreak (ribotype 027)	78
630Δ*erm*	Erythromycin sensitive derivative of *C. difficile* 630	79
AP559	Δ*hexRK*	
AP628	Δ*hexSDF*	
AP654	Δ*hexS*	
AP655	Δ*hexD*	
AP656	Δ*hexF*	
AP237	R20291 pIA34	83
CDE3920	R20291 pCE934	
CDE3921	R20291 pCE935	
AP439	R20291 pCE877	
AP446	R20291 pCE878	
AP441	R20291 pAP114	83
AP572	R20291 pCE942	
AP565	R20291 Δ*hexRK* pAP114	
AP632	R20291 Δ*hexSDF* pAP114	
AP633	R20291 Δ*hexSDF* pCE996	
AP634	R20291 Δ*hexSDF* pCE997	
AP635	R20291 Δ*hexSDF* pCE998	
AP636	R20291 Δ*hexSDF* pCE994	
AP712	R20291 Δ*hexRK* pCE876	
AP546	R20291 pCE876	
AP713	R20291 Δ*hexRK* pCE994	
AP714	R20291 Δ*hexSDF* pCE944	
AP715	630Δ*erm* pIA34	
AP716	630Δ*erm* pCE934	
AP717	630Δ*erm* pCE935	

E. coli strains were grown in LB medium (1% tryptone, 0.5% yeast extract, 0.5% NaCl, and 1.5% agar for solid medium) at 37°C with chloramphenicol at 10 μg/mL and ampicillin at 100 μg/mL as needed.

### Plasmid and bacterial strain construction.

All plasmids are listed in Table 3. Plasmids were constructed using Gibson Assembly (New England Biolabs, Ipswich, MA). Regions of the plasmids constructed using PCR were verified by DNA sequencing. Oligonucleotide primers used in this work were synthesized by Integrated DNA Technologies (Coralville, IA) and are listed in Table S3 in the supplemental material. All plasmids were propagated using OmniMax-2 T1R as a cloning host. CRIPSR-Cas9 deletion plasmids were passaged through E. coli strain MG1655 before transformation into B. subtilis strain BS49 (80). CRISPR-Cas9 plasmids were built on the backbone of pJK02 (81) with some modification (82). CRISPR mutagenesis was performed as previously described (82). Briefly, CRISPR-Cas9, single guide RNA (sgRNA), and 500-bp upstream and downstream homology are provided on a plasmid delivered through conjugation. Genes targeted with sgRNA are subject to double-stranded break via Cas9; homologous recombination is possible through the supplied homology. This recombination even results in a markerless deletion.

**TABLE 3:** Plasmids used in this study

Plasmid	Relevant feature(s)	Reference
pAP24	P*_tet_*-*sLuc^Opt^ catP*	84
pAP114	P*_xyl_*::*mCherryOpt cat*	83
pIA34	P*_xyl_*::*dcas9-opt* P*_gdh_:*:*sgRNA-neg catP*	83
pRPF215	*Ptet-himar1-Ter(slpA)-transposon*	61
pCE876	P*_xyl_*::*hexR^D56E^ catP*	
pCE877	P*_xyl_*::*dcas9-opt* P*_gdh_:*:*hexR-1 catP*	
pCE878	P*_xyl_*::*dcas9-opt* P*_gdh_:*:*hexR-2 catP*	
pCE934	P*_xyl_*::*dcas9-opt* P*_gdh_:*:*hexS-1 catP*	
pCE935	P*_xyl_*::*dcas9-opt* P*_gdh_:*:*hexS-2 catP*	
pCE942	P*_hexS_*-*sLuc^Opt^ catP*	
pCE994	P*_xyl_*::*hexSDF catP*	
pCE996	P*_xyl_*::*hexSF catP*	
pCE997	P*_xyl_*::*hexDF catP*	
pCE998	P*_xyl_*::*hexSD catP*	
pCE918	P*_xyl_*::*cas9-opt* P*_gdh_*::*sgRNA-hexK*	
pCE946	P*_xyl_*::*cas9-opt* P*_gdh_*::*sgRNA-hexS*	
pCE980	P*_xyl_*::*cas9-opt* P*_gdh_*::*sgRNA-hexS*	
pCE999	P*_xyl_*::*cas9-opt* P*_gdh_*::*sgRNA-hexD*	
pCE981	P*_xyl_*::*cas9-opt* P*_gdh_*::*sgRNA-hexF*	

10.1128/mbio.03397-22.9 TABLE S3 Oligonucleotides used in this study. Download Table S3, PDF file, 0.1 MB. Copyright © 2023 Pannullo et al. 2023 Pannullo et al. https://creativecommons.org/licenses/by/4.0/ This content is distributed under the terms of the Creative Commons Attribution 4.0 International license.

For xylose-inducible overexpression constructs, genes of interest were amplified using PCR, and the oligonucleotides are listed in Table S3. PCR amplicons were then inserted into the plasmid pAP114 at the SacI and BamHI sites as described previously (83). For CRISPRi constructs, two guides were created for each targeted gene of interest, and guides were amplified using PCR. The oligonucleotide sequences for the guides are listed in Table S3. PCR amplicons were then inserted into the pIA33 backbone at the MscI and NotI sites as previously described (83). For nanoluciferase reporter constructs, promoters of interest were amplified using PCR; the oligonucleotides are listed in Table S3. PCR amplicons were then inserted into pAP24 (84) at the KpnI and SacI sites using Gibson assembly. These plasmids were then passaged through E. coli HB101/pRK24 for conjugation into C. difficile (85).

### Antibiotic MIC determination.

Overnight cultures of C. difficile were subcultured, grown to late log phase (OD_600_ of 1.0), and then diluted into TY to 10^6^ CFU/mL. For strains containing plasmids with xylose-inducible elements, 1% xylose was added to the overnight cultures and subcultures. A series of antibiotic concentrations were prepared in a 96-well plate in 50 μL TY broth. Wells were inoculated with 50 μL of the diluted late-log-phase culture (0.5 × 10^5^ CFU/well) and grown at 37°C for 16 h. For strains containing vectors with xylose-inducible elements, 1% xylose was included in the media. After the incubation period, the 96-well plates were spun down for 5 min at 5,000 rpm, and the MIC was determined based on the presence of cell pellets. For lysozyme MICs, the steps remained the same, with the exception that after the 16 h incubation, 5 μL from each well was plated on TY for viability. The MIC from this viability plate was read the following day, with the MIC being defined as the lowest concentration of lysozyme at which 5 or fewer colonies were found per spot.

### Nanoluciferase assays.

Overnight cultures of C. difficile were subcultured 1:100 and grown until mid-log phase (OD_600_, ~0.5). For antibiotic induction experiments, a series of antibiotic concentrations were prepared in a 96-well plate in 50 μL TY broth. Wells were inoculated with 50 μL of mid-log-phase culture. Inoculated plates were allowed to incubate for 2 h at 37°C in the anaerobic chamber. Fifty microliters from each well was then transferred to solid white 96-well plates. A 1:100 dilution was made of the Nano-Glo luciferase assay (Promega) working solution. Fifty microliters of the diluted Nano-Glo solution was added to each well in the white plate. Plates were allowed to rest at room temperature for 5 min prior to luminescence measurement. Luminescence was measured using an Infinite M200 Pro plate reader (Tecan). Luminescence values were normalized to OD_600_.

### RNA extraction.

Overnight cultures were grown in TY and then subcultured 1:100 in 100 mL of TY. Subcultures were grown to an OD_600_ of 0.7 to 0.8, mixed 1:1 with a solution of 50% ethanol and 50% acetone, and immediately frozen at −80°C. Samples were stored at −80°C until they were ready for harvest. Extraction of RNA was performed using the RNeasy extraction kit (Qiagen) as previously described (54). The RNA integrity number (RIN) was determined using the Agilent Bioanalyzer 2000 at the University of Iowa.

### RNA-seq and analysis.

Sequencing was performed by (SeqCenter, Pittsburgh, PA) using 51-bp paired-end (PE) reads. We achieved a read depth of ~80 to 120× per sample. Data analysis was done using the ProkSeq (86) pipeline, which performs trimming and quality control (QC), and sequence alignment using Bowtie2, quantification of RNA expression levels using FeatureCounts, and differential expression analysis using DEseq2 (87). The full RNA-seq data set can be found in Table S2.

### Lipidomics.

Overnight cultures were grown in TY supplemented with 1% xylose and 10 μg/mL thiamphenicol in biological triplicate. Overnight cultures were then subcultured to an OD_600_ of 0.05 in TY supplemented with 1% xylose and 10 μg/mL thiamphenicol. Subcultures were allowed to grow until an OD_600_ of 0.6 to 0.7 was reached, at which point the cells were harvested and pelleted at 8,000 rpm for 20 min. Biological replicates (3) were grown on different days.

Lipid extraction and LC-MS/MS were performed by Cayman Chemical Company. After thawing, cells were mixed with 5 mL methanol, transferred to 7-mL Precellys tubes containing 0.1-mm ceramic beads (Bertin Technologies; CK01 lysing kit), and homogenized with 3 cycles at 8,800 rpm for 30 s, with 60-s pauses between cycles. Then, 800 μL of the homogenized mixtures was transferred to 8-mL screw-cap glass tubes. A methyl *tert*-butyl ether (MTBE)-based liquid-liquid extraction protocol was used by first adding 1.2 mL methanol containing a mixture of deuterated internal standards covering several major lipid categories (fatty acids, glycerolipids, glycerophospholipids, sphingolipids, and sterols) and then 4 mL MTBE. The mixture was incubated on a tabletop shaker at 500 rpm at room temperature for 1 h and then stored at 4°C for 60 h to maximize lipid extraction. After bringing the samples to room temperature, phase separation was induced by adding 1 mL water to each sample. The samples were vortexed and then centrifuged at 2,000 × *g* for 15 min. The upper organic phase of each sample was carefully removed using a Pasteur pipette and transferred into a clean glass tube. The remaining aqueous phase was reextracted with 2 mL of the upper phase of MTBE/methanol/water at 10:3:2.5 (vol/vol/vol). After vortexing and centrifuging, the organic phase was collected and combined with the initial organic phase. The extracted lipids were dried overnight in a SpeedVac vacuum concentrator.

The dried lipid extracts were reconstituted in 200 μL *n*-butanol–methanol at 1:1 (vol/vol) and transferred into autosampler vials for analysis by LC-MS/MS. Aliquots of 5 μL were injected into an Ultimate 3000 ultraperformance liquid chromatography (UPLC) system connected to a Q Exactive Plus Orbitrap mass spectrometer (Thermo Scientific). An Accucore C_30_ 2.6 μm, 150- by 2.1-mm HPLC column (Thermo Scientific) was used, using mobile phases A (acetonitrile/water/formic acid 60:40:0.1 [vol/vol/vol], containing 10 mM ammonium formate) and B (acetonitrile/isopropanol/formic acid 10:90:0.1 [vol/vol/vol], containing 10 mM ammonium formate). Lipids were eluted at a constant flow rate of 300 μL/min using a gradient from 30% to 99% mobile phase B over 30 min. The column temperature was kept at a constant 40°C. Polarity switching was used throughout the gradient to acquire high-resolution MS data (resolution, 75,000) and data-dependent MS/MS data.

Data analysis was performed using Lipostar software (version 2; Molecular Discovery) for detection of features (peaks with unique *m/z* and retention time), noise and artifact reduction, alignment, normalization, and lipid identification. Automated lipid identification was performed by querying the Lipid Maps Structural Database (LMSD), modified by Cayman to include many additional lipids not present in the LMSD.

Statistical analysis of the data presented in Fig. 4 was performed on the summed peak areas of lipids with the same head groups and normalized to the WT values to allow for comparison between strains. Data were analyzed with one-way analysis of variance using Dunnett’s multiple-comparison test.

### Transposon mutagenesis of C. difficile.

We generated a pooled transposon library in R20291 consisting of ~80,000 colonies using pRPF215 as previously described (61). Briefly, mid-log-phase C. difficile R20291 containing pRPF215 was plated on TY supplemented with 20 μg/mL lincomycin and 50 ng/mL anhydrotetracycline. After overnight growth, we pooled the library. To identify genes required for daptomycin resistance, we grew the pooled transposon library with or without 0.5 μg/mL daptomycin. We chose 0.5 μg/mL daptomycin, as this is a sublethal concentration compared to WT C. difficile (MIC of ~4 μg/mL), and we hypothesized that this sub-MIC level would be a suitable cutoff to find insertions that led to greatly increased sensitivity. Briefly, 100 μL of the prepared transposon library was subcultured into 5 mL of TY supplemented with 20 μg/mL lincomycin and 100 ng/mL anhydrotetracycline. Subcultures were grown until an OD_600_ of 0.2 to 0.3 was reached, at which point 0 or 2 μg/mL daptomycin was added. Cultures were incubated with daptomycin until OD_600_ 1.0 was reached; cells were then pelleted. Genomic DNA (gDNA) was extracted by washing cell pellets 3 times in 1× PBS. Pellets were suspended in 300 μL of 1× ThermoPol buffer (New England Biolabs), and 5 μL of 800 U/mL proteinase K (New England Biolabs) was added. Cells were incubated in solution at 37°C for 1 h until they were clearly lysed. The DNA was extracted using phenol/chloroform/isoamyl alcohol (25:24:1; Fischer Scientific Company) followed by ethanol precipitation of the DNA. The purified gDNA was resuspended in 100 μL of double-distilled water (ddH_2_O).

### Sequencing analysis of transposon insertions.

Preparation of the transposon amplicon library was performed as previously described (88). Briefly, the primer CDEP4815 was used to perform multiple rounds of single-primer extension. The extension products were then C-tailed using terminal transferase (New England Biolabs). The resulting C-tailed extension products were then amplified using primers CDEP4816 and P1 or CDEP4816 and P3 to generate barcoded amplicon libraries for Illumina sequencing. Fragments of 300 to 500 bp were extracted from a gel and purified. Sequencing was performed on Illumina HiSeq X with 150-bp PE reads. Sequencing was performed by Admera Health (South Plainfield, NJ).

The resulting read data were analyzed using the Transit package for Tn-seq analysis (89). Briefly, the reads were demultiplexed using Sabre (90). The data were then trimmed and aligned to eliminate poor-quality reads using the TRANSIT preprocessor (89). TRANSIT aligned the trimmed reads to the C. difficile R20291 genome using the Burrows-Wheeler Aligner (BWA), tabulated Tn insertion counts, and calculated conditional essentially utilizing the resampling comparison method (89).

### Data availability.

RNA-seq data were submitted to the NCBI GEO repository and assigned accession number GSE220119.
