# Supplementary figures and images for: HexSDF Is Required for Synthesis of a Novel Glycolipid That Mediates Daptomycin and Bacitracin Resistance in C. difficile
Source: mBio. 2023 Feb 14;14(2):e03397-22. doi: 10.1128/mbio.03397-22 (PMC10128005; doi:10.1128/mbio.03397-22)

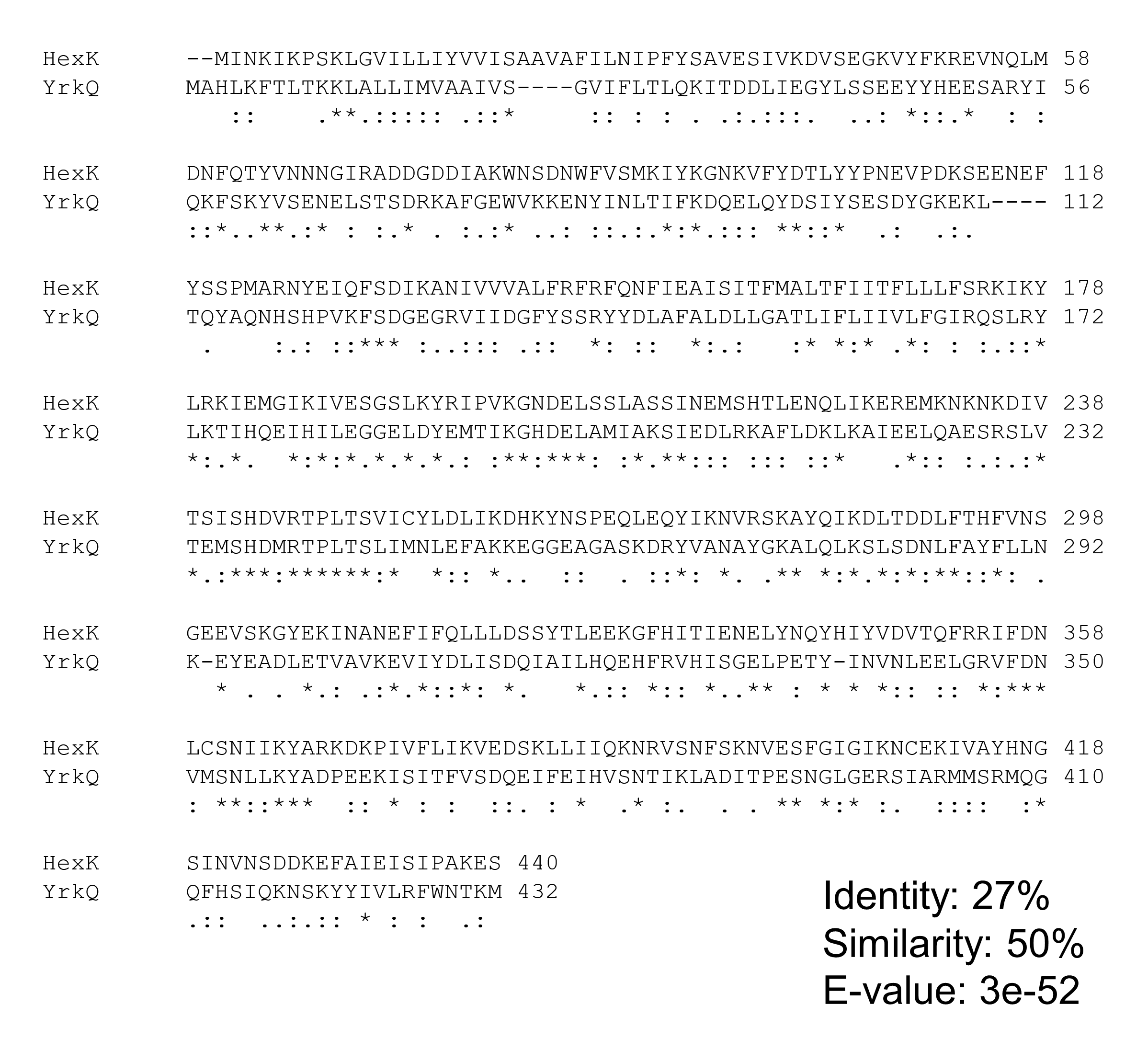

Supplement: FIG S1 [file mbio.03397-22-s0004.tif]

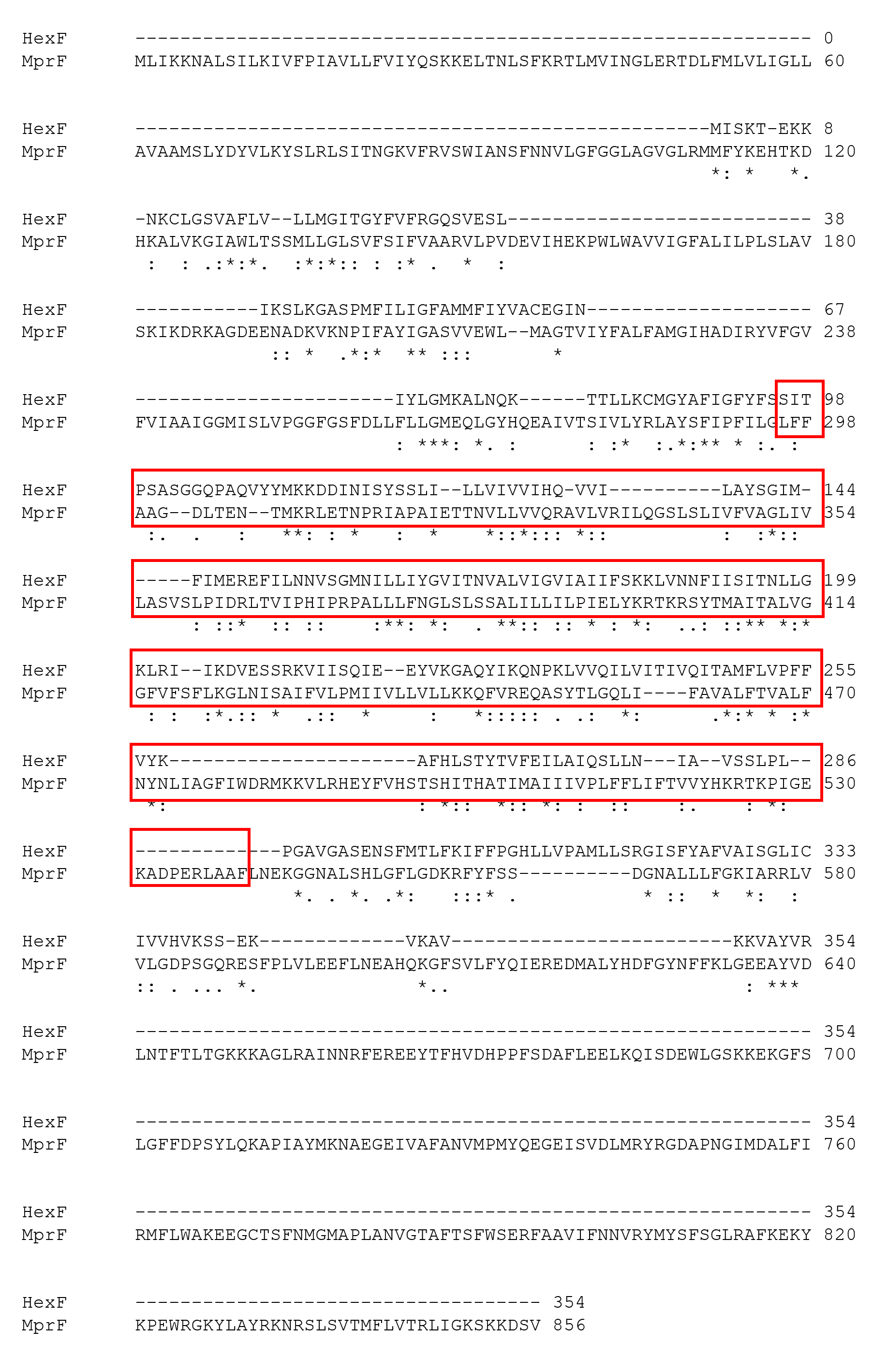

Supplement: FIG S2 [file mbio.03397-22-s0005.tif]

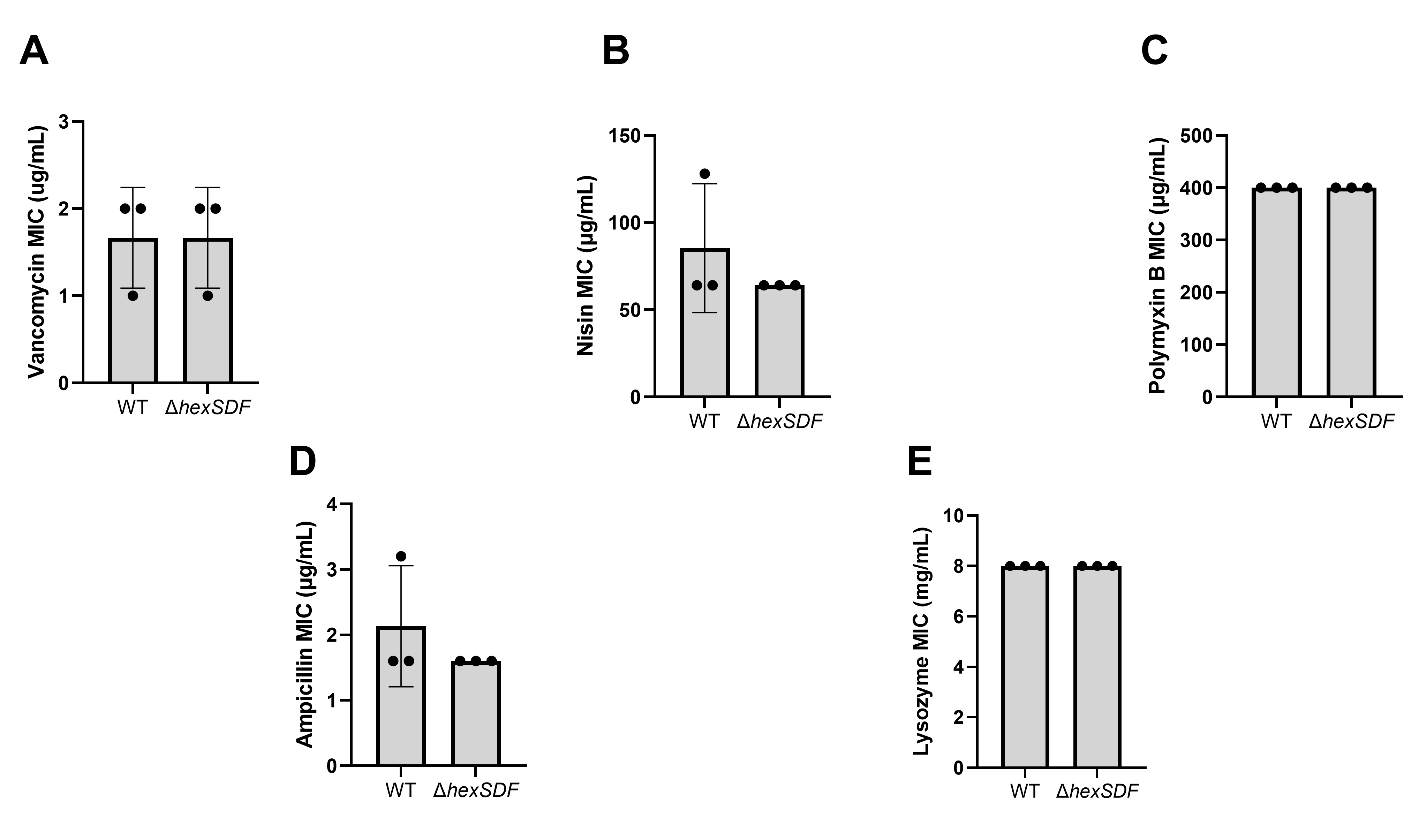

Supplement: FIG S3 [file mbio.03397-22-s0006.tif]

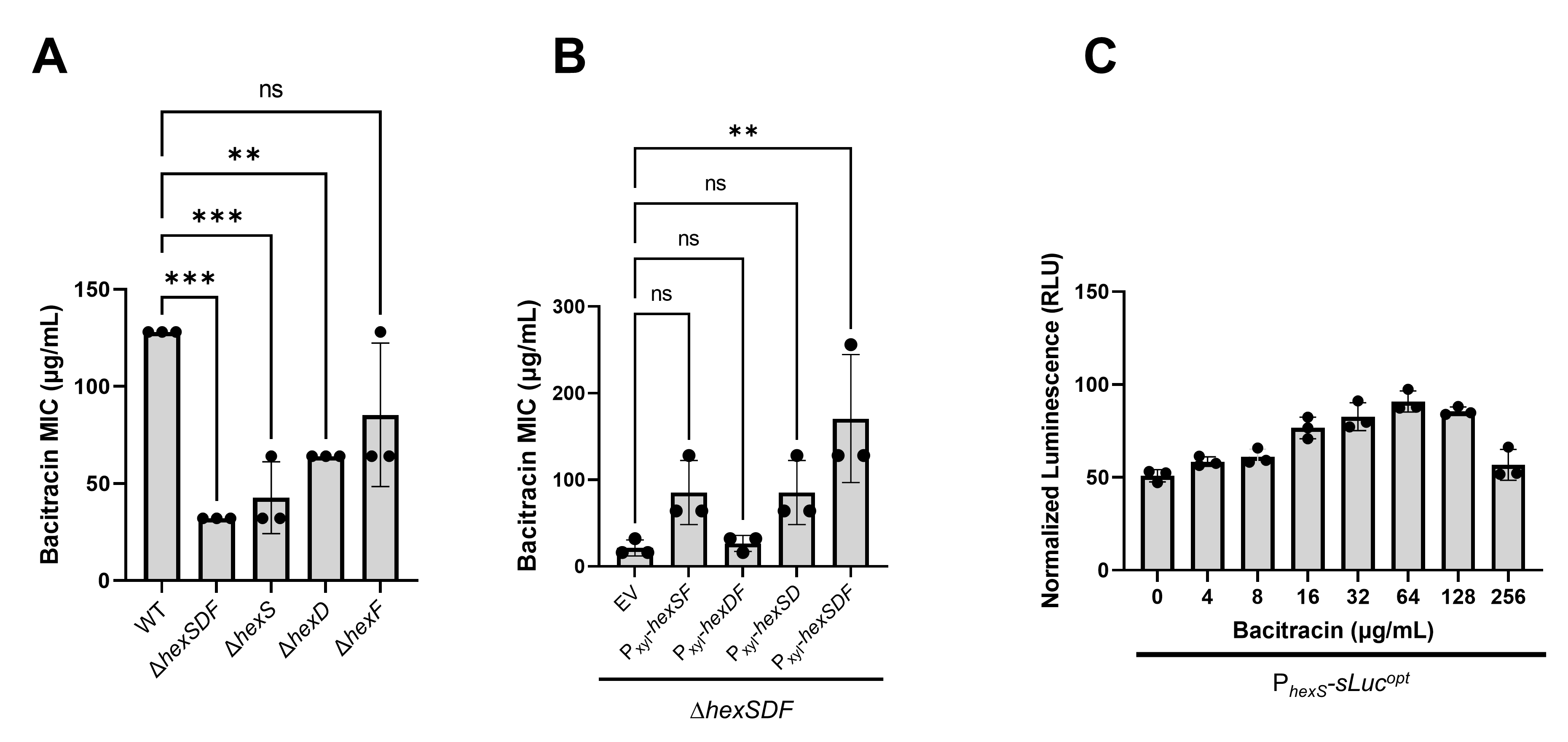

Supplement: FIG S4 [file mbio.03397-22-s0007.tif]

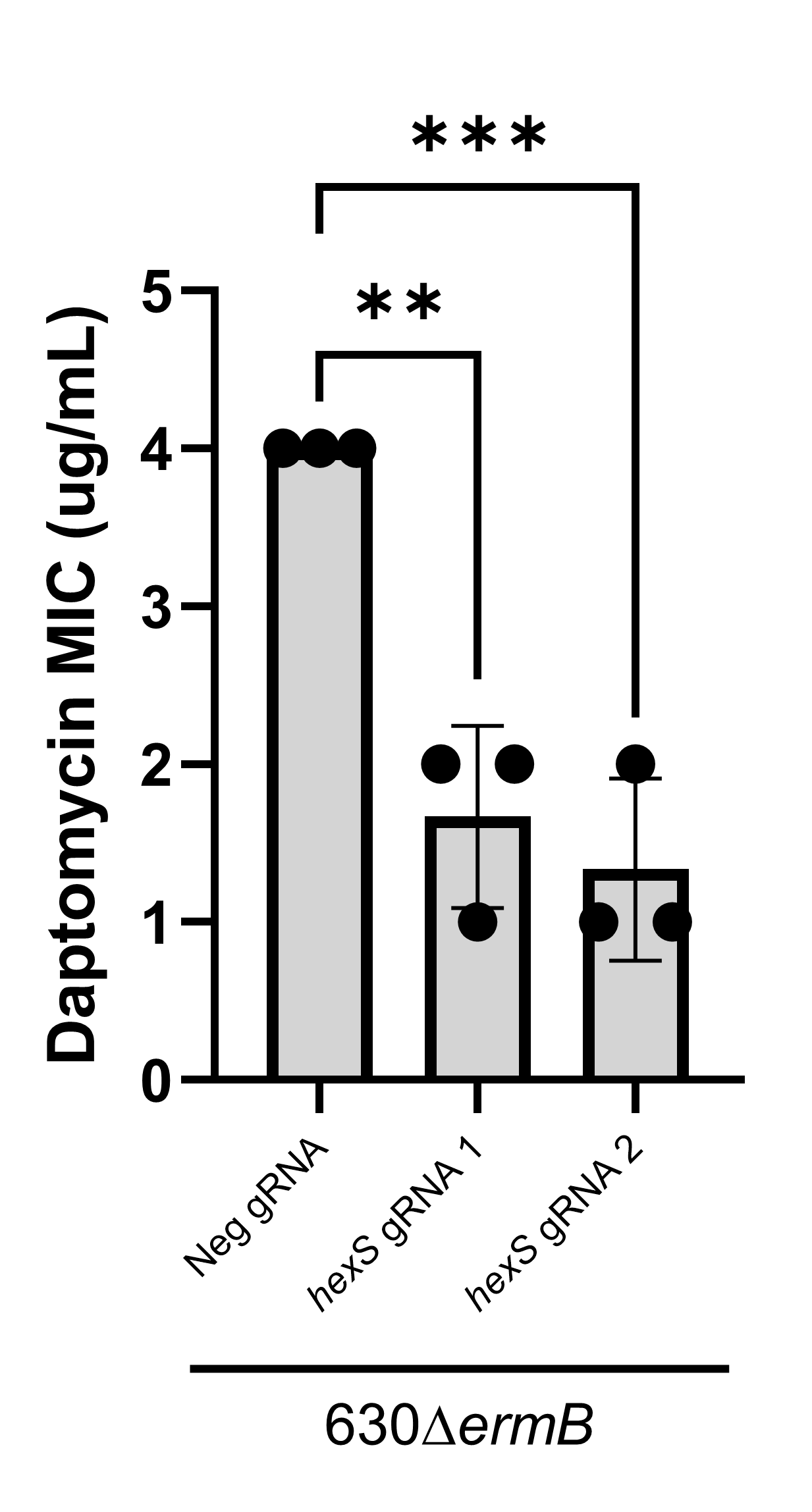

Supplement: FIG S5 [file mbio.03397-22-s0008.tif]

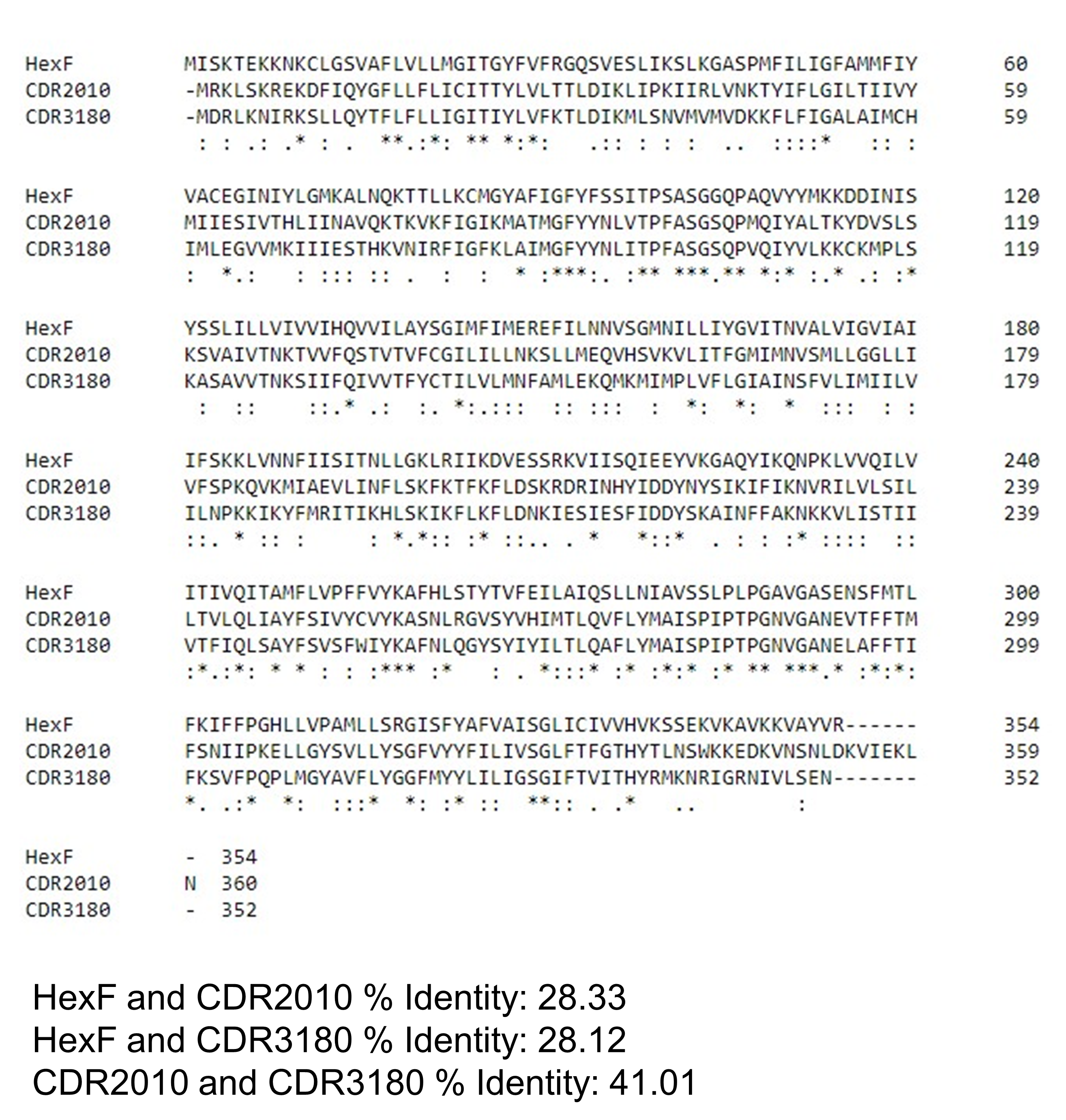

Supplement: FIG S6 [file mbio.03397-22-s0009.tif]
