# Supplementary material for: HexSDF Is Required for Synthesis of a Novel Glycolipid That Mediates Daptomycin and Bacitracin Resistance in C. difficile
Source: mBio. 2023 Feb 14;14(2):e03397-22. doi: 10.1128/mbio.03397-22 (PMC10128005; doi:10.1128/mbio.03397-22)
Supplement: TABLE S1 [file mbio.03397-22-s0001.pdf]

Table S1 - TnSeq Statsitics of the *hex* Cluster

Gene Name	Log2FC	P-Value (FDR-Corrected)
hexK	-4.46	0.0076
hexR	-1.6	0.0558
hexF	-2.63	0.5587
hexD	-7.59	0.0579
hexS	-0.61	0.7218
