# Supplementary material for: HexSDF Is Required for Synthesis of a Novel Glycolipid That Mediates Daptomycin and Bacitracin Resistance in C. difficile
Source: mBio. 2023 Feb 14;14(2):e03397-22. doi: 10.1128/mbio.03397-22 (PMC10128005; doi:10.1128/mbio.03397-22)
Supplement: TABLE S3 [file mbio.03397-22-s0003.pdf]

Table S3. Oligonucleotides

Oligo	Sequence	Relevant features
CDEP5259	aattaaactgtaaatggcca TCTACGATTTCCCTAATCTC<br>GTTTTAGAGCTAGAAATAGC	*sgRNA-hexR-1*
CDEP5260	aattaaactgtaaatggcca ATTAGTTCAGAATATGAAAA<br>GTTTTAGAGCTAGAAATAGC	*sgRNA-hexR-2*
CDEP5593	aattaaactgtaaatggccaTACATTTTATCTGAATAGTT<br>gttttagagctagaaatagc	*sgRNA-hexS-1*
CDEP5594	aattaaactgtaaatggccaAAAGTTGAAATAACTACTGT<br>gttttagagctagaaatagc	*sgRNA-hexS-2*
CDEP5296	aaacagctatgaccgcggccAGTCATAACTATTGTACAAATAAC<br>AGC	Upstream *hexRK*
CDEP5297	ACACAATATATAATTTAAATTTCACCAAGCTCCTTTTTTT<br>GCT	Upstream *hexRK*
CDEP5298	GCAAAAAAAGGAGCTTGGTGAAATTTAAATTATATATTG<br>TGTAATTGCTATTATATATT	Downstream *hexRK*
CDEP5299	TTATTTTTATGCTAGctcgaGAACTAACATAACAAATCCAG<br>TTAC	Downstream *hexRK*
CDEP5332	aattaaactgtaaaggtacc TAGTAAAAGATGTATCAGAA<br>GTTTTAGAGCTAGAAATAGC	*sgRNa-hexK*
CDEP3876	aaccatctaaaaatagttgcagagcttACGCGTC	Cloning into pJK02 derivatives
CDEP5633	aaacagctatgaccgcggccCTGGTAAAGCAGCAGAAAAG	Upstream *hexSDF*
CDEP5634	aACTTTCAGTTTAGCGGTCTGGGCGCCAAGATTCCCTC<br>CTCGTTAAAGCTTTTC	Upstream *hexSDF*
CDEP5635	GGCGCCCAGACCGCTAAACTGAAAGTtTATTATATAGAT<br>TATTTTTATTAATATGTCAATTTGTG	Downstream *hexSDF*
CDEP5636	TTATTTTTATGCTAGctcgaCTCTTTTGTTTTTAATATATTT<br>GGCTC	Downstream *hexSDF*
CDEP5651	aattaaactgtaaatggcca TTTTCTATTCTTGTAAATCG<br>GTTTTAGAGCTAGAAATAGC	*sgRNA-hexS*
CDEP5649	aattaaactgtaaatggcca AACTACTATCGTCCACCATG<br>GTTTTAGAGCTAGAAATAGC	*sgRNA-hexD*
CDEP5650	aattaaactgtaaatggcca CCGGAACTAATAAGTGACCA<br>GTTTTAGAGCTAGAAATAGC	*sgRNA-hexF*
CDEP5771	GGCGCCCAGACCGCTAAACTGAAAGTtTAGGTTTAATTA<br>TCGTTGTTGCATTGA	Clone homology for *hexS* deletion
CDEP5772	TTATTTTTATGCTAGctcgaGGTGCACCTACTGCACCACC	Clone homology for *hexS* deletion

CDEP5773	aaacagctatgaccgcggccATGAAGGTATTAATTCTTACAGGAAAATTTG	Clone homology for *hexD* deletion
CDEP5774	aACTTTCAGTTTAGCGGTCTGGGCGCCTCTCTACACGCTCCTTGTTCATCAAGT	Clone homology for *hexD* deletion
CDEP5775	GGCGCCCAGACCGCTAAACTGAAAGTtTTATGATAAGTAAAACTGAAAAGAAAAATAA	Clone homology for *hexD* deletion
CDEP5776	TTATTTTTATGCTAGctcgaTTATCTTACATAAGCAACTTTTTTCA	Clone homology for *hexD* deletion
CDEP5769	aaacagctatgaccgcggccGGCTGGAGAAAATGAAATTGC	Clone homology for *hexF* deletion
CDEP5770	aACTTTCAGTTTAGCGGTCTGGGCGCCAActaCATCCTTTCTGGTGTTACGAAC	Clone homology for *hexF* deletion
CDEP5626	gcttcttatttttatggtacAGAACATTGGTTGTTGGTTT	clone P_{hexS} into pAP24 digested w/ KpnI and SacI
CDEP5627	tctcctttactgcaggagctTAAGATTCCCTCCTCGTTAAAG	clone P_{hexS} into pAP24 digested w/ KpnI and SacI
CDEP5624	CGATAGTTATGAAGTGAGCTTAAGGAGGGGAATCTTATGAAGGTATTAATTCTTAC	clone *hexSDF* onto pAP114
CDEP5625	TCTATTTAAAGTTTTATTAAAACTTATAGGATCTTAATTAAAATGTACACACAAATTGACA	clone *hexSDF* onto pAP114
CDEP5799	TTATTAAAACTTATAGGATCCTACATCCTTTCTGGTGTTACG	clone *hexSD* onto pAP114
CDEP5800	CGATAGTTATGAAGTGAGCTTAAGGAGGTGTAGAGAATGTATGTAGTAGGTTTAAT	clone *hexDF* onto pAP114
CDEP5801	TTCAGTTTTACTTATCATAACTACATACATTCTCTACACGCT	Clone *hexSF* onto pAP114
CDEP5802	CGTGTAGAGAATGTATGTAGTTATGATAAGTAAAACTGAAAAGAAAAATAA	Clone *hexSF* onto pAP114
CDEP5265	CGATAGTTATGAAGTGAGCTGCAAAAAAAGGAGCTTGGTG	Clone *hexR* onto pAP114
CDEP5266	TTTATTAAAACTTATAGGATCGGCTTAATTTTGTTAATcaaTATAATACCCC	Clone *hexR* onto pAP114
CDEP5267	GATTGATTTGATTATTTTGGAaGTTGTAATGCCTGTAAAAACAGG	Clone *hexR* D56E
CDEP5268	CCTGTTTTTACAGGCATTACAACtTCCAAAATAATCAAATCAATC	Clone *hexR* D56E
CDEP4815	atcactccttcttaattacaaatttttagcatctaatttaaciiiiittcctattatac	Transpson library single primer extension

CDEP4816	CAAGCAGAAGACGGCATACGAGCTCTTCCGATCTGGG<br>GGGGGGGGGGGGG	Transposon library universal<br>barcode primer
P1	AATGATACGGCGACCACCGAGATCTACACTCTTTCCCT<br>ACACGACGCTCTTCCGATCT NNNNAG ATCACG<br>ctgtcagaccggggacttatcagccaacct	Transpson library barcode P1
P3	AATGATACGGCGACCACCGAGATCTACACTCTTTCCCT<br>ACACGACGCTCTTCCGATCT NNNNAG TGACCA<br>ctgtcagaccggggacttatcagccaacct	Transpson library barcode P3
